# The normobaric oxygen paradox: does it increase haemoglobin?

**DOI:** 10.1186/cc9842

**Published:** 2011-03-11

**Authors:** S Theunissen, D De Bels, J Devriendt, P Germonpré, P Lafere, J Valsamis, T Snoeck, P Meeus, C Balestra

**Affiliations:** 1 ISEK Environmental Physiology Laboratory, Brussels, Belgium; 2 Brugmann University Hospital, Brussels, Belgium; 3 Queen Astrid Military Hospital, Brussels, Belgium

## Introduction

A novel approach to increase erythropoietin (EPO) using oxygen has been reported in healthy volunteers. The purpose of this study is to investigate whether the EPO increase is sufficient to induce erythropoiesis.

## Methods

We compared exposure to daily versus every other day oxygen administration on haemoglobin variation during a 12-day period. Each subject underwent the two protocols at a 6-week interval period to achieve the same baseline values.

## Results

**Figure 1:**
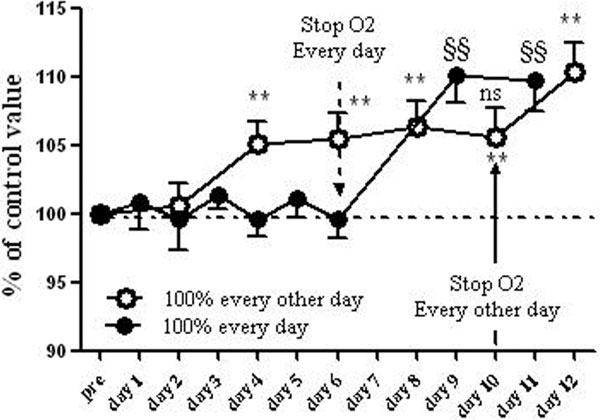
**Comparison between haemoglobin variations after 30 minutes of 100% O2 breathing every day or every other day**. **Statistically significant difference from baseline (*P *< 0.01) for oxygen breathing every other day (protocol B). ^§§^Statistically significant difference from baseline (*P *< 0.01) for Oxygen breathing each day (protocol A).

See Figure 1. Nine subjects underwent the study. We observed a significant increase in haemoglobin values in the every other day group compared with the each day group and with baseline. At the end of each day period, haemoglobin values increased to achieve a significant difference as compared with baseline. There was a significant rise of reticulocytes in the every other day group as compared with the each day group (182 ± 94% vs. 93 ± 34%, *P *< 0.001). These data provide demonstration of an enhanced production of erythrocytes.

## Conclusions

The normobaric oxygen paradox seems effective to increase haemoglobin in non-anaemic healthy volunteers assuming there is a sufficient time interval between the two oxygen applications. This could permit interesting clinical applications in perioperative medicine as an adjunct therapy to EPO for blood predonation.

